# *Pseudomonas aeruginosa* Elastase Contributes to the Establishment of Chronic Lung Colonization and Modulates the Immune Response in a Murine Model

**DOI:** 10.3389/fmicb.2020.620819

**Published:** 2021-01-12

**Authors:** Cristina Cigana, Jérôme Castandet, Nicolas Sprynski, Medede Melessike, Lilha Beyria, Serena Ranucci, Beatriz Alcalá-Franco, Alice Rossi, Alessandra Bragonzi, Magdalena Zalacain, Martin Everett

**Affiliations:** ^1^Division of Immunology, Transplantation and Infectious Diseases, IRCCS San Raffaele Scientific Institute, Milan, Italy; ^2^Antabio SAS, Labège, France; ^3^Nurix Therapeutics, San Francisco, CA, United States

**Keywords:** Pseudomonas aeruginosa, elastase, LasB, murine chronic lung infection, immune response, cystic fibrosis

## Abstract

Chronic infection by *Pseudomonas aeruginosa* in cystic fibrosis (CF) patients is a major contributor to progressive lung damage and is poorly treated by available antibiotic therapy. An alternative approach to the development of additional antibiotic treatments is to identify complementary therapies which target bacterial virulence factors necessary for the establishment and/or maintenance of the chronic infection. The *P. aeruginosa* elastase (LasB) has been suggested as an attractive anti-virulence target due to its extracellular location, its harmful degradative effects on host tissues and the immune system, and the potential to inhibit its activity using small molecule inhibitors. However, while the relevance of LasB in acute *P. aeruginosa* infection has been demonstrated, it is still unclear whether this elastase might also play a role in the early phase of chronic lung colonization. By analyzing clinical *P. aeruginosa* clonal isolates from a CF patient, we found that the isolate RP45, collected in the early phase of persistence, produces large amounts of active LasB, while its clonal variant RP73, collected after years of colonization, does not produce it. When a mouse model of persistent pneumonia was used, deletion of the *lasB* gene in RP45 resulted in a significant reduction in mean bacterial numbers and incidence of chronic lung colonization at Day 7 post-challenge compared to those mice infected with wild-type (wt) RP45. Furthermore, deletion of *lasB* in strain RP45 also resulted in an increase in immunomodulators associated with innate and adaptive immune responses in infected animals. In contrast, deletion of the *lasB* gene in RP73 did not affect the establishment of chronic infection. Overall, these results indicate that LasB contributes to the adaptation of *P. aeruginosa* to a persistent lifestyle. In addition, these findings support pharmacological inhibition of LasB as a potentially useful therapeutic intervention for *P. aeruginosa*-infected CF patients prior to the establishment of a chronic infection.

## Introduction

*Pseudomonas aeruginosa* is a Gram-negative opportunistic pathogen with a remarkable and threatening ability to thrive and adapt to various ecological niches (Stover et al., 2000). It causes significant morbidity and mortality, in particular in patients with acute nosocomial airway infections, such as ventilator-associated pneumonia, and with chronic respiratory diseases, including cystic fibrosis (CF), non-CF bronchiectasis, and chronic obstructive pulmonary disease (COPD; Emerson et al., 2002; Jacobs et al., 2020). The high incidence of these infections is ascribable to the large arsenal of virulence factors (e.g., proteases, pyocyanin, exotoxins) secreted by *P. aeruginosa*, which enables it to cause damage to the host and overcome host immune responses, thus promoting infection (Galdino et al., 2019b). The zinc metalloprotease elastase B (also called LasB or pseudolysin) is the most abundant protease in the *P. aeruginosa* secretome (Galdino et al., 2019b). Expression of this protease is regulated in a cell population density-dependent manner through cell-to-cell communication, i.e., “quorum sensing” (QS), and, after processing in the periplasm, the mature protein is secreted by the type II secretion system (T2SS; Michel et al., 2011). LasB can degrade components of the extracellular matrix, such as elastin, collagen, fibronectin, and mucins and components of the cellular junctions, such as vascular endothelial cadherin, inducing tissue injury, and bacterial dissemination (Golovkine et al., 2014; Flynn et al., 2017; Galdino et al., 2019b). It can also alter epithelial integrity and repair efficiency (Ruffin and Brochiero, 2019), thus favoring the spread of infection. In addition, LasB can inactivate components of the complement system, such as C3 (Bastaert et al., 2018), degrade other components of the immune defenses (e.g., interleukin 6 – IL-6; Saint-Criq et al., 2018), and interfere with bacterial killing by alveolar macrophages (Kuang et al., 2011; Bastaert et al., 2018), thus manipulating the host response to *P. aeruginosa*.

In the context of chronic respiratory diseases, *lasB* is expressed in the airway secretions of CF patients (Storey et al., 1992) and individuals with COPD during exacerbations (Murphy et al., 2008), suggesting a potential role in the progression of lung disease. Recently, LasB has also been shown to reduce expression of the CF transmembrane conductance regulator (CFTR) and its activity in epithelial cells *in vitro* (Saint-Criq et al., 2018). Defective CFTR functionality has been associated with failure to eradicate inhaled bacteria (Cohen and Prince, 2012); thus, it is conceivable that CFTR impairment by LasB could favor the establishment of chronic *P. aeruginosa* colonization not only in CF patients but also in patients with COPD and non-CF bronchiectasis. In this regard, deletion of *lasB* was shown to lead to a less invasive infection in a mouse model of acute pneumonia when compared to the infection caused by the respective wt strain (Golovkine et al., 2014), confirming the relevance of LasB in pathogenesis of *P. aeruginosa* infection. The most common animal model used so far to assess the relevance of LasB reproduces acute respiratory infection (Bastaert et al., 2018; Qu et al., 2018; Saint-Criq et al., 2018), but a chronic infection model has not been previously used. Thus, it is not clear whether and how LasB is involved in the establishment of chronic colonization.

In this study, we investigated the impact of LasB in chronic *P. aeruginosa* lung colonization with the aim to (1) determine whether LasB plays a role in the establishment of chronic lung colonization in a mouse model and (2) establish how LasB affects the host immune response.

## Materials and Methods

### Ethics Statement

Animal studies adhered to the Italian Ministry of Health guidelines for the use and care of experimental animals (IACUC #879). Research with *P. aeruginosa* isolates from CF individuals and storage of biological materials were approved by the Ethics Commission of Hannover Medical School, Germany.

### Bacterial Strains

*P. aeruginosa* strains included the reference strain PAO1 (Cigana et al., 2020), as well as CF isolate RP45 (Jeukens et al., 2013; Bianconi et al., 2015) and its late pathoadaptive variant RP73 isolated 7 years after RP45 from the same CF patient (Bianconi et al., 2015; Cigana et al., 2016a, 2020).

### Construction of *lasB*-Deleted (∆*lasB*) Mutants in *P. aeruginosa* PAO1, RP45, and RP73 Strains

The *lasB* gene was deleted by homologus recombination according to the method reported by Kaniga et al. (1991). Upstream and downstream regions (700 bp in length) flanking *lasB* were obtained by gene synthesis (GeneArt®) and cloned into the suicide vector pKNG101 as a BamHI/SpeI fragment. These regions were specific for strains PAO1, RP45, and RP73 as some variations were observed. *Escherichia coli* CC118*λ*pir was used as host for pKNG101 plasmids. The recombinant plasmids were further transferred into *P. aeruginosa* strains by conjugation and transconjugants were selected on Pseudomonas isolation agar (PIA) plates containing streptomycin at 2,000 μg/ml. Negative selection was further performed on Luria-Bertani (LB) medium supplemented with 5% sucrose. Allelic exchanges were screened by PCR, and deletion of the1,497 bp *lasB* gene was confirmed by sequencing.

### Elastolytic Assay (Elastin Congo Red Method) to Evaluate LasB Activity in *P. aeruginosa* Strains

This assay measures the elastolytic activity of secreted LasB in the supernatant of *P. aeruginosa* cultures using elastin congo red (ECR, Sigma) as substrate. LasB degrades elastin and releases the congo red dye into the supernatant which can then be measured spectrophotometrically. Overnight cultures of *P. aeruginosa* (PAO1, RP45, and RP73) wt and ∆*lasB* strains were diluted in LB medium. After reaching an OD_600nm_ of 0.6, cultures were diluted 1:100 and incubated for an additional 24 h in a shaking incubator. The culture supernatants were recovered by centrifugation and filtered through a 0.22 μm filter. The supernatants (diluted 1:10 in LB medium) were then mixed 1:1 with 2X ECR solution, resulting in a final concentration of 10 mg/ml ECR in 50 mM Tris-HCl pH 7.4 and 0.5 mM CaCl_2_, and incubated for 20 h in a 37°C shaking incubator. The supernatants were recovered by centrifugation, and the released congo red dye was quantitated by its absorbance at 495 nm (OD_495nm_). As a negative control, supernatants were replaced by LB medium. Statistical analyses were performed according to Student *t*-test using values obtained with LB medium as reference.

### Mouse Model of Chronic Lung Infection

C57BL/6NCrlBR male mice (8–10 weeks, Charles River) were challenged by intratracheal (i.t.) administration with an average of 5.6 × 10^5^ colony forming units (CFUs) of the wt and ∆*lasB* RP45 strains or 3 × 10^5^ CFUs of the wt and ∆*lasB* RP73 strains embedded in agar-beads (Bragonzi et al., 2009; Facchini et al., 2014; Cigana et al., 2016b) or with sterile empty agar-beads as control. Body weight and health status were monitored daily. Lung CFUs were analyzed at Days 2 and 7 post-infection. As reported previously, recovery of ≥1,000 CFUs of *P. aeruginosa* from Day 7 lung cultures is considered evidence that a chronic infection has been established (Lore et al., 2016, 2018). Cytokine/chemokine/growth factor levels were measured in the supernatant of lung homogenates by Bio-Plex Assay (Bio-Rad), as described in the online data supplement. Additional details in accordance with the ARRIVE guidelines (Kilkenny et al., 2010) are reported in the online data supplement.

### Statistics

Statistical analyses were performed with GraphPad Prism (GraphPad Software, Inc., San Diego, CA, USA), using Student *t*-test for the elastolytic activity, a two-way analysis of variance (ANOVA) with Bonferroni’s multiple comparison test for body weight changes, Fisher’s exact test for the incidence of chronic colonization and survival, and nonparametric two-tailed Mann-Whitney test for the CFUs and levels of chemokines/cytokines/growth factors. Outlier data, identified by Grubbs’ test, were excluded from the analysis. A value of *p* < 0.05 was considered to be statistically significant.

## Results

### LasB Production and Activity Differ Among *P. aeruginosa* Strains

**Figure 1:**
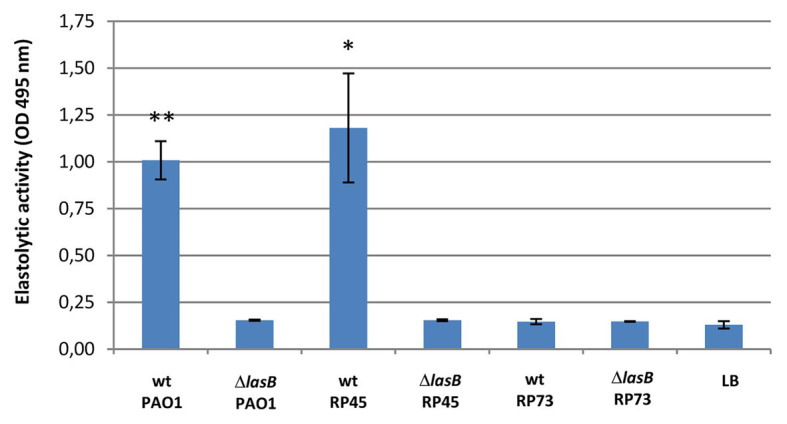
LasB elastolytic activity in culture supernatants from *Pseudomonas aeruginosa* PAO1 and cystic fibrosis (CF) isolates RP45 and RP73 [wild-type (wt) vs. ∆*lasB* mutants]. LasB activity in bacterial supernatants (prepared by centrifugation and filtration after 24 h of growth and diluted to 1:10) was evaluated by Elastolytic assay (ECR method, after 20 h of incubation with ECR substrate). Luria-Bertani (LB) growth medium was used as negative control. The data were pooled from two independent experiments in quadruplicate (*n* = 8 per strain). Statistical significance is indicated as follows: ^*^p < 0.05; ^**^p < 0.01.

The LasB elastolytic activities of culture supernatants from *P. aeruginosa* reference strain PAO1 and clinical isolates RP45 and RP73, collected from a CF patient at different periods following the onset of lung colonization, were investigated by the ECR method (Figure 1). Whereas the RP45 isolate displayed a strong LasB activity (*p* = 0.007 for PAO1 vs. LB medium, *p* = 0.036 for RP45 vs. LB medium), the later clonal variant RP73 (isolated 7 years after RP45) showed no elastolytic activity, despite the presence of LasB protein being confirmed in cultures supernatants by Western blot (Supplementary Figure S1A). The loss of LasB activity is in accordance to the pathoadaptation of *P. aeruginosa* previously observed for some strains during development of chronic infection in CF (Bianconi et al., 2015). Since all three strains (PAO1, RP45, and RP73) have identical *lasB* sequences, the lack of activity in the RP73 variant is possibly due to incorrect processing of the mature protein. As expected, all the corresponding ∆*lasB* mutants displayed no elastolytic activity (Figure 1).

### LasB Promotes Chronic *P. aeruginosa* Lung Colonization

**Figure 2:**
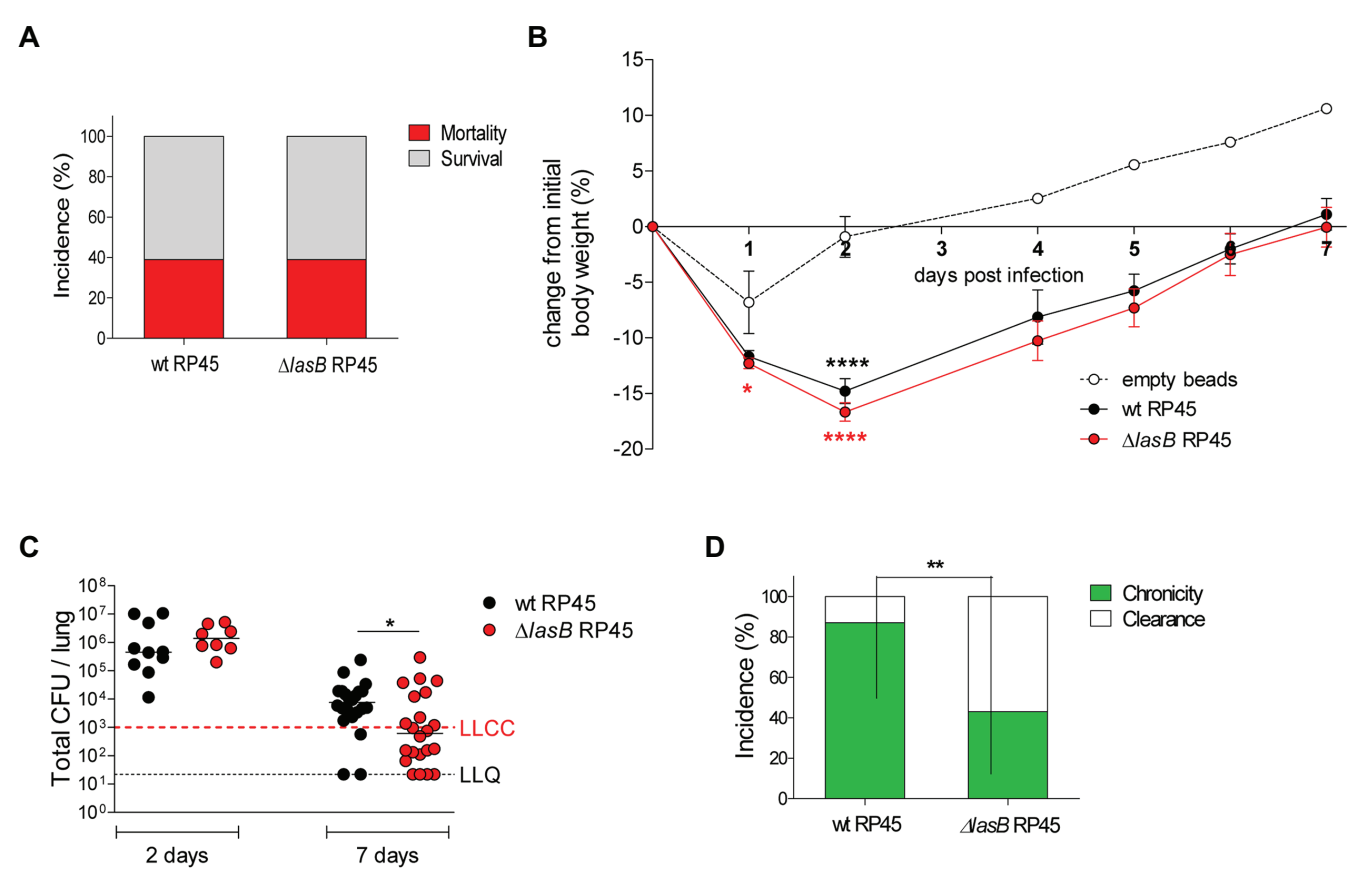
Impact of chronic lung infection by wt and ∆*lasB* mutant *P. aeruginosa* RP45 strains on host response. C57BL/6NCrlBR mice were infected with an average of 5.6 × 10^5^ colony forming units (CFUs) embedded in agar beads or with sterile empty beads. Mice were monitored daily for survival and body weight. At Days 2 and 7 post-infection, mice were euthanized, and lungs were excised and homogenized. **(A)** The incidences of survival and mortality were determined on challenged mice and expressed as percentage. **(B)** Body weight data are expressed as mean ± standard error of the mean (SEM) of the weight change from the initial body weight. **(C)** CFUs were evaluated in the lungs after plating onto tryptic soy agar at Days 2 and 7 post-infection. Dots represent values of individual mice, and horizontal lines represent median values. The lower limit of chronic colonization (LLCC) and the lower limit of quantification (LLQ) are indicated. **(D)** The incidence of clearance (<1,000 *P. aeruginosa* CFUs in the lung cultures) and capacity to establish chronic airways infection (≥1,000 *P. aeruginosa* CFUs in the lung cultures) were determined on surviving mice at Day 7 post-infection and expressed as percentage. The data were pooled from three independent experiments (mice *nr* = 38 per strain). Statistical significance is indicated as follows: ^*^p < 0.05; ^**^p < 0.01; ^****^p < 0.0001.

**Figure 3:**
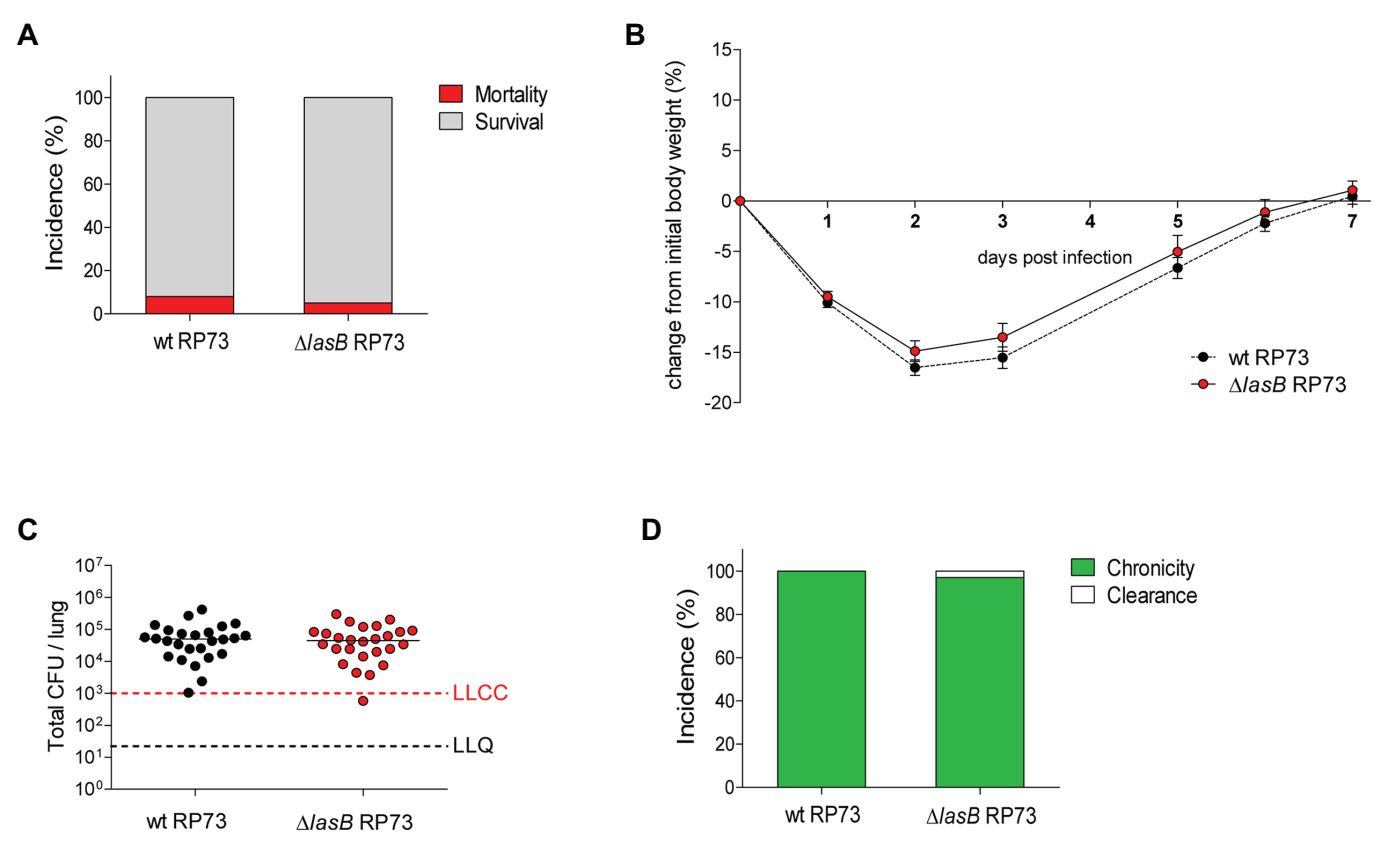
Impact of chronic lung infection by wt and ∆*lasB* mutant *P. aeruginosa* RP73 strains on host response. C57BL/6NCrlBR mice were infected with an average of 3 × 10^5^ CFUs/lung embedded in agar beads. Mice were monitored daily for survival and body weight. At Day 7 post-infection, mice were euthanized, and lungs were excised and homogenized. **(A)** The incidences of survival and mortality were determined on challenged mice and expressed as percentage. **(B)** Body weight data are expressed as mean ± standard error of the mean (SEM) of the weight change from the initial body weight. **(C)** CFUs were evaluated in the lungs after plating onto tryptic soy agar at Day 7 post-infection. Dots represent values of individual mice, and horizontal lines represent median values. The lower limit of chronic colonization (LLCC) and the lower limit of quantification (LLQ) are indicated. **(D)** The incidence of clearance (<1,000 CFUs of *P. aeruginosa* in the lung cultures) and capacity to establish chronic airways infection (≥1,000 CFUs of *P. aeruginosa* in the lung cultures) were determined on surviving mice at Day 7 post-infection and expressed as percentage. The data were pooled from three independent experiments (mice *nr* = 28–29).

In order to investigate the role of LasB in chronic lung colonization by *P. aeruginosa*, a ∆*lasB* mutant was generated for the RP45 strain. Mice were infected with wt or ∆*lasB* RP45 bacteria embedded in agar beads and monitored daily for body weight and health status. Identical rates of survival (60%) were observed for mice infected with the wt RP45 strain and those infected with the ∆*lasB* mutant counterpart (Figure 2A; Supplementary Figure S2). Both infected groups of animals lost significantly more body weight than mice challenged with sterile empty beads, although no differences were observed in the loss or recovery of body weight between mice infected with wt or ∆*lasB* RP45 strains (Figure 2B). At Day 2 post-infection, the lung bacterial burden did not differ between mice infected with wt or ∆*lasB* RP45 strains. However, at Day 7 post-infection, mice infected with the ∆*lasB* RP45 strain showed significantly lower mean lung CFUs than those infected with the wt counterpart (*p* = 0.0329, Figure 2C). This reflected the higher capacity of clearance of RP45 ∆*lasB* (61%) vs. the wt strain (14%), which resulted in a lower incidence of chronic colonization (*p* = 0.0045, Figure 2D). This suggests that LasB is involved in the establishment of chronic colonization in this model. Mice infected with the wt RP45 strain harboring the highest bacterial load at Day 2 post-infection had detectable LasB by Western blot in lung homogenates (Supplementary Figure S1B) although enzymatic activity could not be detected in these samples (data not shown), perhaps due to the presence of inhibitors in the homogenate and/or limitations in the assay sensitivity. In addition, a ∆*lasB* mutant was generated for the late adapted strain RP73. The survival rate of mice infected with wt RP73 strain was similar to that of mice infected with ∆*lasB* RP73 bacteria (≈90%; Figure 3A) but higher than that of mice infected with wt RP45 strain, as previously shown (Bianconi et al., 2015). No difference in changes in body weight was observed between wt or ∆*lasB* RP73-infected mice (Figure 3B). The lung bacterial burden in mice infected with wt RP73 strain was similar to that in mice infected with ∆*lasB* RP73 strain (≈5 × 10^4^ median CFUs; Figure 3C) but higher than that in mice infected with wt RP45 bacteria (≈7.7 × 10^3^ median CFUs). When the incidence of chronic colonization was analyzed, no difference was observed between mice infected with wt or ∆*lasB* RP73 bacteria (Figure 3D), probably due to the absence of LasB activity in RP73. Overall, these results indicate that LasB can contribute to the establishment of chronic lung colonization in CF patients, but its activity could be dispensable in strains already well-adapted to a chronic infection lifestyle.

### LasB Affects Inflammation Induced by Chronic *P. aeruginosa* Lung Colonization

In order to evaluate whether LasB could affect inflammation, the levels of several chemokines/cytokines and growth factors were measured in the supernatants of lung homogenates from mice infected with wt or ∆*lasB* RP45 strains at Day 7 post-infection. Four cytokines/chemokines (IL-5, IL-10, IL-13, and granulocyte-macrophage colony-stimulating factor – GM-CSF) were undetectable in the majority of the samples; thus, they were excluded from further analysis. The ∆*lasB* RP45 strain induced significantly higher levels of IL-1α, IL-1β, IL-3, IL-12p70, IL-17A, eotaxin, granulocyte colony-stimulating factor (G-CSF), macrophage inflammatory protein 1α (MIP-1α), MIP-1β, and RANTES in comparison to the wt counterpart (Table 1), indicating that LasB can downregulate inflammation. These differences did not correlate with the bacterial burden, which is not surprising since mice infected with the ∆*lasB* RP45 strain showed higher levels of clearance in comparison to those infected with wt RP45. When the analysis was limited to those mice still colonized at Day 7 post-infection, the ∆*lasB* RP45 strain induced significantly higher levels of IL-1α, IL-1β, IL-9, IL-12p70, G-CSF, MIP-1α, MIP-1β, RANTES, and tumor necrosis factor (TNF)-α in comparison to the wt RP45 strain (Supplementary Table S1), confirming the immunomodulatory potential of LasB.

**Table 1:** Cytokine/chemokine concentrations following chronic airway infection by wt and *ΔlasB* RP45 strains.

Cytokine/Chemokine	Concentration (mean pg/700 μg lung protein ± SEM)	
	wt RP45 strain	Δ*lasB* RP45 strain
IL-1α	7,36 ± 1,12	14,66 ± 1,53^***^
IL-1*β*	3,08 ± 0,33	5,35 ± 0,33^****^
IL-2	6,26 ± 0,28	6,20 ± 0,37
IL-3	0,92 ± 0,05	1,11 ± 0,05^*^
IL-4	0,52 ± 0,03	0,60 ± 0,04
IL-6	3,75 ± 0,06	3,54 ± 0,16
IL-9	18,47 ± 0,51	19,78 ± 0,69
IL-12p40	34,05 ± 2,37	38,89 ± 1,82
IL-12p70	18,21 ± 1,19	24,71 ± 1,42^***^
IL-17A	11,37 ± 1,00	15,73 ± 1,29^*^
Eotaxin	574,0 ± 23,22	640,6 ± 18,07^*^
G-CSF	10,36 ± 0,95	21,10 ± 2,00^****^
IFN-γ	20,59 ± 0,80	22,29 ± 0,43
KC	63,71 ± 5,08	73,22 ± 6,69
MCP-1	169,0 ± 11,38	169,9 ± 6,84
MIP-1α	14,64 ± 2,45	40,57 ± 4,21^****^
MIP-1β	36,08 ± 3,80	76,55 ± 7,44^****^
RANTES	248,0 ± 21,83	392,4 ± 40,96^*^
TNF-α	31,58 ± 1,12	34,91 ± 1,15

Statistical significance is indicated. C57BL/6NCrlBR male mice (8 to 10 weeks old) were infected intratracheally (i.t.) with an average of 5.6 × 10^5^ colony forming units of the *Pseudomonas aeruginosa* wt and ΔlasB RP45 strains embedded in agar-beads. After 7 days, murine lungs were collected, homogenized, and centrifuged. The supernatants were used to measure cytokine/chemokine levels by Bio-Plex assay. Data are expressed as mean values ± standard errors of the mean (SEMs) of results from mice pooled from three independent experiments (*n* = 18–19). Statistical significance is indicated with

* *p* < 0.05

*** *p* < 0.001

**** *p* < 0.0001.

## Discussion

*P. aeruginosa* infections pose a major threat to patients with chronic respiratory infections, such as those with CF or COPD (Emerson et al., 2002; Jacobs et al., 2020). The metalloprotease LasB, the most abundant protein in the *P. aeruginosa* secretome (Saint-Criq et al., 2018; Galdino et al., 2019b), has been shown to interfere with several pathophysiological pathways. In addition to causing tissue injury and manipulating the immune response (Golovkine et al., 2014; Bastaert et al., 2018; Saint-Criq et al., 2018; Galdino et al., 2019b; Ruffin and Brochiero, 2019), it also reduces the expression and activity of CFTR (Saint-Criq et al., 2018). This evidence assumes considerable relevance as LasB-producing *P. aeruginosa* strains have been commonly isolated in CF sputum secretions (Storey et al., 1992, 1997; Tingpej et al., 2007). However, most of the evidence supporting the role of LasB is based on *in vitro* studies or animal models of acute infection. In this work, we exploited the mouse model of chronic lung infection, involving the i.t. inoculation of bacteria embedded in agar-beads, to evaluate the effect of LasB on the establishment of chronic colonization. Agar-beads have been shown to reproduce a microaerobic environment that allows bacteria to grow in the form of microcolonies, similar to growth in the mucus of CF patients (Bragonzi et al., 2005). Two clonal *P. aeruginosa* clinical isolates, collected from a CF patient at different stages of chronic infection, and their corresponding ∆*lasB* mutants were used in these studies. We had previously demonstrated that the RP45 strain induced mortality in half of infected mice and caused chronic infection in approximately 80% of the surviving mice, whereas the RP73 strain, isolated 7 years after RP45, possessed several pathoadaptive traits, in that it was non-lethal and exhibited an extraordinary capacity to establish a long-term chronic lung infection (colonizing almost all infected mice; Bianconi et al., 2015). Here, we show that deletion of *lasB* in the RP45 strain did not affect the rate of survival of infected mice or their body weight as a parameter of mouse health. Surviving mice, including those chronically colonized, recovered body weights, suggesting the establishment of an equilibrium between bacterial growth and containment by the host response. However, despite similar percent survival and loss of body weight, a key finding was that deletion of *lasB* resulted in a lower incidence of chronic lung colonization, indicating that LasB may play a key role in the early stage of the adaptation of *P. aeruginosa* to chronic colonization. Interestingly, no difference in the bacterial load, between wt and ∆*lasB* RP45 infected mice, was observed in those animals with an established chronic infection at Day 7 (defined as those harboring ≥1,000 CFUs/lung), further indicating that the role of LasB may be restricted to an early establishment of chronic infection rather than maintenance of the chronic state.

No difference was observed in the incidence of chronic colonization between mice infected with wt or ∆*lasB* RP73 strains at Day 7 post-infection. The wt RP73 strain does not produce an active LasB protein. The high incidence of chronic infection is consistent with the RP73 strain being isolated from a patient with a long-term chronic lung infection and thus well adapted to a chronic infection lifestyle in the CF lung. Indeed, *P. aeruginosa* undergoes a well-known evolutionary adaptation during long-term colonization in the CF lung, which is sustained by bacterial lineages clonal to the initially acquired strain and carrying pathoadaptive phenotypic traits. The phenomenon of adaptation to the CF lung allows bacteria to face a complex environment, characterized by a thick and viscous mucus, in which intense selective pressures are exerted by components of the host immune response, variable availability of nutrients and oxygen, and continuous antibiotic treatments (Rossi et al., 2020). RP73 strain has been shown to possess several features of adaptation to the CF airways that make it highly efficient in chronically colonizing the host (Bianconi et al., 2015). The fact that RP73 no longer requires an active LasB protein in order to cause chronic infection further supports the hypothesis that LasB may only play a role in the early adaptation to a persistent lifestyle and subsequent chronic infection rather than in its maintenance. These results are consistent with works reporting that the LasB expression and activity are more frequently described in early stage sputum isolates compared to those from chronic infections in CF patients (Tingpej et al., 2007; Harmer et al., 2013). A potential mechanistic explanation for this is offered by the observations that (i) LasB expression is required for degradation of complex substrates, such as mucins, during early infections when nutrients are in short supply (Flynn et al., 2017) and (ii) LasB expression is suppressed under anaerobic conditions associated with severe and chronic infections (Lee et al., 2011). Thus, LasB may be important for establishing an early (aerobic) infection, through tissue invasion, degradation of host immune response factors, and supply of growth substrates but is then surplus to requirement during subsequent chronic (anaerobic) infection, characterized by compromised host defenses and abundant free amino-acids.

Analyses of the inflammatory response in the murine lung show that deletion of *lasB* increased cytokines, chemokines, and growth factors is associated with both innate and adaptive immune responses. In particular, *lasB* deletion was associated with an increase in the production of factors related to the recruitment of neutrophils (e.g., GM-CSF, MIP-1α), monocytes/macrophages (e.g., MIP-1β), natural killer cells (e.g., IL-12p70, MIP-1β) and T cells (e.g., RANTES), or differentiation of T helper 1 (e.g., IL-12p70) and T helper 17 cells (e.g., IL-17A). This indicates that LasB can cause immunomodulation by either direct degradation of immune mediators or indirect downregulation of their production. However, in contrast to the data reported by Saint-Criq and colleagues (Saint-Criq et al., 2018), we did not observe a downregulation of IL-6 in this study. This could be explained by the timing of the analysis (in addition to the other variables such as strain, mouse model, and inoculum size); whereas Saint-Criq and colleagues measured IL-6 levels after 24 h of acute infection, we analyzed concentrations 7 days after inoculation in the mouse model of chronic lung infection. The cytokine levels observed did not positively correlate with the bacterial loads, which may be because mice infected with the ∆*lasB* RP45 strain showed higher levels of clearance than those infected with wt RP45. We hypothesize that the increased immune response facilitated by deletion of *lasB* may have contributed to the higher incidence of bacterial clearance. Further studies, based on the analysis of cellular recruitment and on the inhibition or stimulation of specific immune components, in particular in the early phases of infection/colonization, may be helpful to dissect further the molecular mechanism underlying LasB-mediated immunomodulation. Others have demonstrated that LasB is able to compromise various host immune response factors and that LasB deficiency or inhibition can lead to increased survival or reductions in bacterial burden in mouse models of acute lung infection when a non-lethal bacterial inoculum has been used (Kuang et al., 2011; Bastaert et al., 2018; Qu et al., 2018; Saint-Criq et al., 2018); however, to our knowledge, this is the first study to address the impact of LasB on the incidence of chronic colonization. Future studies could further corroborate its impact on chronic colonization using additional *P. aeruginosa* clonal lineages.

Overall, our findings indicate that LasB can compromise the immune response, thereby promoting the persistence of *P. aeruginosa* in the lung. These data support ongoing investigations of LasB-targeted therapeutic approaches to reduce the establishment of chronic *P. aeruginosa* colonization (Galdino et al., 2019a).

## Data Availability Statement

The raw data supporting the conclusions of this article will be made available by the authors, without undue reservation.

## Ethics Statement

The animal study was reviewed and approved by IRCCS San Raffaele Scientific Institute (Milan, Italy), Institutional Animal Care and Use Committee (IACUC #733 and #878) and adhered strictly to the Italian Ministry of Health guidelines for the use and care of experimental animals.

## Author Contributions

CC, AB, NS, MZ, and ME contributed to conception and design of the study and interpreted the data. CC, MM, SR, BA-F, and AR performed *in vivo* experiments and collected data. CC and JC analyzed the data, performed statistical analysis, and prepared the figures and tables. JC and LB performed Western blot and evaluated LasB activity. CC wrote the first draft of the manuscript. AB, MZ, and ME critically revised the manuscript. All the authors read, revised and approved the final manuscript.

### Conflict of Interest

The authors declare that this study received funding from Antabio SAS (France) with financial support from Cooperative Agreement Number IDSEP160030 from ASPR/BARDA and by awards from Wellcome Trust and Germany’s Federal Ministry of Education and Research, as administrated by CARB-X. Antabio had the following involvement in the study: study concept and design, interpretation of data, writing of the article. CARB-X was not involved in the study design, collection, analysis, and interpretation of data, the writing of this article or the decision to submit it for publication. The content is solely the responsibility of the authors and does not necessarily represent the official views of the Department of Health and Human Services Office of the Assistant Secretary for Preparedness and Response, other funders, or CARB-X.

Author SR was employed by company Nurix Therapeutics. The remaining authors declare that the research was conducted in the absence of any commercial or financial relationships that could be construed as a potential conflict of interest.
