# Supplementary material for: Pseudomonas aeruginosa Elastase Contributes to the Establishment of Chronic Lung Colonization and Modulates the Immune Response in a Murine Model
Source: Front Microbiol. 2021 Jan 12;11:620819. doi: 10.3389/fmicb.2020.620819 (PMC7836092; doi:10.3389/fmicb.2020.620819)
Supplement: Supplementary file 1 [file Data_Sheet_1.pdf]

SUPPLEMENTARY DATA

SUPPLEMENTARY MATERIALS AND METHODS

Mouse model. Immunocompetent C57BL/6NCrlBR male mice (8-10 weeks of age) were purchased from Charles River (Calco, Italy), shipped in protective, filtered containers, transported in climate-controlled trucks, and allowed to acclimatize for at least two days in the animal house prior to use. Mice were maintained in sterile ventilated cages in the biosafety level 3 (BSL3) facility at San Raffaele Scientific Institute (Milano, Italia) where 3-5 mice per cage were housed. Mice were fed with standard rodent autoclaved chow (VRFI, Special Diets Services, UK) and autoclaved tap water. Fluorescent lights were cycled 12h on, 12h off, and ambient temperature (23±1°C) and relative humidity (40-60%) were regulated.

For infection experiments, mice were anesthetized by an intraperitoneal injection of a solution of Avertin (2,2,2- tribromethanol, 97%) in 0.9% NaCl and administered at a volume of 0.015 ml/g body weight. Mice were placed in supine position. The trachea was directly visualised by ventral midline, exposed and intubated with a sterile, flexible 22-g cannula attached to a 1 ml syringe. An inoculum of 50-70 µl of agar-bead suspension was implanted via the cannula into the lung. After inoculation, all incisions were closed by suture.

Mice were monitored daily for coat quality, posture, attitude, ambulation, hydration status and body weight. Mice that lost >20% body weight and had evidence of severe clinical disease, such as scruffy coat, inactivity, loss of appetite, poor locomotion, or painful posture, were humanely euthanized before the termination of the experiments with an overdose of carbon dioxide. Gross lung pathology was noted.

Mice were humanely euthanized two and seven days post-infection. Lungs were excised aseptically and homogenized in 2 ml PBS containing protease inhibitors (Complete tablets, Roche Diagnostic) using the homogenizer gentleMACS™ Octo Dissociator. 100 µl of the undiluted homogenates and 10-fold serial dilutions were spotted onto tryptic soy agar (TSA). Colony Forming Units (CFUs) were determined after overnight growth at 37°C. The remaining lung homogenate was centrifuged at 16,000 *g*, 30 min, 4°C, and the supernatant collected and stored at -80°C.

Infections and euthanasia were all performed in the late morning. In addition, in all the experiments, mice had been subdivided according to the body weight to have similar mean in all the groups of infection.

Animal studies were conducted according to protocols approved by San Raffaele Scientific Institute (Milan, Italy) Institutional Animal Care and Use Committee (IACUC #733 and #878) and adhered strictly to the Italian Ministry of Health guidelines for the use and care of experimental animals.

Agar-beads preparation for chronic infection. The agar-beads *P. aeruginosa* mouse model was used (Bragonzi et al., 2009; Facchini et al., 2014; Cigana et al., 2016). Aliquots of glycerol stocks of *P. aeruginosa* RP45 or RP73 wt and *lasB* deleted mutant (Δ*lasB*) strains were streaked on tryptic soy agar (TSA) plates and incubated at 37°C O/N. One colony from each plate was used to inoculate 10 ml of tryptic soy broth (TSB) and placed in a shaking incubator at 37°C 200 rpm O/N. The O/N bacterial suspension was diluted to 0.1 OD/ml for RP45 strains or 0.15 OD/ml for RP73 strains in 20 ml of TSB / flask and grown for 3 h for RP45 strains or 4 h for RP73 strains at 37°C at 200rpm, to reach the log phase. The bacteria were pelleted by centrifugation (2,700 *g*, 15 min, 4°C) and resuspended in 1 ml PBS (pH 7.4). A total of 2 x10^9 *P. aeruginosa* CFUs were used for inclusion in the agar-beads, prepared according to previously described methods (Bragonzi et al., 2009; Facchini et al., 2014; Cigana et al., 2016). Bacteria were added to 9 ml of 1.5% TSA (w/v), prewarmed to 50°C. As a control, sterile empty agar-beads were prepared by the same procedure, except that 1 ml of sterile PBS, rather than bacteria, was added to 9 ml of 1.5% TSA (w/v), prewarmed to 50°C. These mixtures were pipetted forcefully into 150 ml heavy mineral oil/each at 50°C and stirred rapidly with a magnetic stirring bar for 6 min at room temperature, followed by cooling at 4°C with continuous slowly stirring for 35 min, and then left at 4°C for 15 min. The oil-agar mixtures were centrifuged at 2,700 *g* for 15 min to sediment the beads, which were washed six times in PBS at room temperature. The size of the beads was verified microscopically and only those preparations containing beads of

100 µm to 200 µm in diameter were used as inoculum for animal experiments. The number of *P. aeruginosa* CFUs in the beads was determined by plating serial dilutions of the homogenized bacteria-bead suspension on TSA plates. The inoculum was prepared by diluting the bead suspension with PBS to inoculate about $5x10^5$ CFU/50-70µl. *P. aeruginosa* beads were prepared the day before inoculation and stored overnight at 4°C. The number of *P. aeruginosa* CFUs in the inoculated beads was determined by plating serial dilutions of the homogenized bacteria-bead suspension at the day of the infection. Sterile empty agar-beads were diluted at the same density of the solution containing agar-beads-embedded bacteria.

Gel electrophoresis and Western blot analysis to evaluate LasB levels in culture supernatants and lung homogenates. SDS polyacrylamide gel electrophoresis (SDS-PAGE) and Western blotting were carried out on culture supernatants of wt and Δ*lasB* mutant *P. aeruginosa* PAO1, RP45 and RP73 and lung homogenates from infected mice to demonstrate LasB production in these samples. The culture supernatants were prepared as described in the Elastolytic Assay method, and diluted 1:10 in LB. The lung homogenates were prepared by centrifugation at 6,000 rpm for 5 min followed by filtration of the supernatants (0.2 µm). Filtrates were mixed with Laemmli 2X sample buffer (Sigma), boiled 5 min at 100°C and used for analysis by gel electrophoresis and Western blot. The samples were loaded onto a 12% Mini Protean TGX precast protein gel (Bio-Rad) for culture supernatants and onto a 4-15% Mini Protean TGX precast protein gel (Bio-Rad) for the lung homogenates, and the proteins were separated on the gel by electrophoresis at 200 V constant for 40 min. After migration, the gel was transferred to polyvinylidene difluoride (PVDF) membrane (Bio-Rad) for Western blot analysis, using a Bio-Rad Trans-Blot Turbo Transfer System, for 3 min at a constant setting of 2.5 A and voltage fixed at up to 25 V. Non-specific binding sites were blocked by 1h incubation with slow shaking at room temperature with 5% skimmed milk protein in Tris-buffered saline buffer containing 0.1% Tween (TBS-T buffer: 20 mM Tris-HCl pH 7.4, 150 mM NaCl, 0.1% Tween 20). An antibody against *P. aeruginosa* elastase LasB was provided by E. Kessler (Tel Aviv University, Israel). After transfer, membranes were labeled for 1 h incubation with slow shaking at room temperature using anti-LasB rabbit polyclonal primary antibody prepared at 1:50,000 in TBS-T buffer with 0.5% skimmed milk protein. Membranes were then washed three times with TBS-T with 0.5% skimmed milk protein, for 5 min each time, before being reprobed with goat anti-rabbit alkaline phosphatase-conjugated secondary antibody (Sigma), prepared at 1:30,000 in TBS-T buffer with 0.5% skimmed milk protein, for 1 h incubation with slow shaking at room temperature and washed three more times with TBS-T for 5 min each time. Finally, membranes were incubated with NBT/BCIP (Sigma), chromogenic substrate for alkaline phosphatase for 5 to 30 min to visualize the LasB protein.

Quantification of cytokine/chemokine/growth factor concentration. The protein content in the supernatants of murine lung homogenates was determined by the Bradford assay (Bio-RAD). The Bio-Plex pro Mouse Cytokine Standard 23-Plex (Bio-Rad) based on Luminex technology, was used for the quantification of cytokines, chemokines and growth factors (IL-1α, IL-1β, IL-2, IL-3, IL-4, IL-5, IL-6, IL-9, IL-10, IL-12 (p40), IL-12 (p70), IL-13, IL-17A, Eotaxin, G-CSF, GM-CSF, IFN-γ, KC, MCP-1 (MCAF), MIP-1α, MIP-1β, RANTES, TNF-α) in the supernatants of murine lung homogenates, according to the manufacturer's instruction. Results were expressed as pg/700µg protein of lung homogenate supernatant. Data were measured on Bio-Plex 200 System and calculated using Bio-Plex Manager 6.0 and 6.1 software.

SUPPLEMENTARY FIGURES

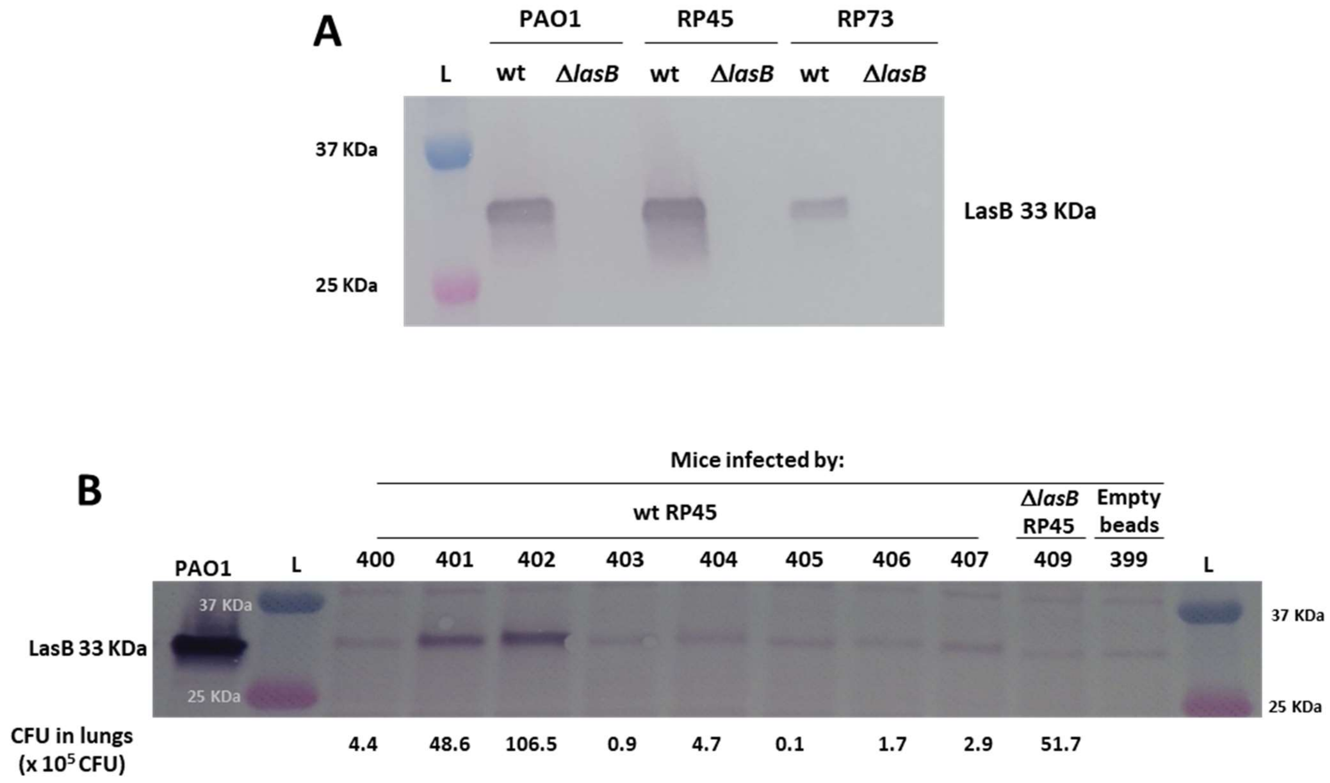

Figure S1: **LasB production in bacterial culture supernatants and lung samples. A)** Supernatants were recovered from 24h-cultures of PAO1, RP45 and RP73 wt and Δ*lasB* strains. **B)** Ten lung samples from infected mice, recovered at Day 2 post-infection, were analyzed by Western blot (8 samples for wt RP45, 1 for Δ*lasB* RP45 strain and 1 for sterile empty agar-beads). Supernatant of *P. aeruginosa* PAO1 was used as reference and positive control. Lung sample from mice inoculated with empty agar-beads was used as negative control. Bacterial loads expressed as CFU in lungs (x 10^5) are noted. LasB protein was visualized with rabbit polyclonal antibody against *P. aeruginosa* elastase and an alkaline phosphatase-conjugated secondary antibody. Migration position of the 33 KDa band corresponds to mature LasB protein. L, ladder.

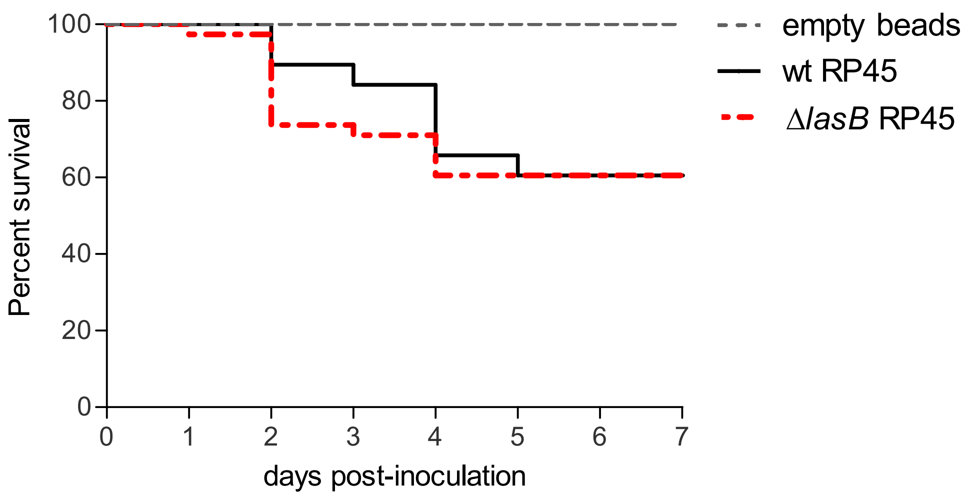

Figure S2: Impact of chronic lung infection by *P. aeruginosa* RP45 wt and Δ*lasB* mutant strains on mouse survival. C57BL/6NCrlBR mice were infected with an average of 5.6×10^5 CFU/lung embedded in agar beads or with sterile empty beads and monitored daily for survival. Survival data are expressed as percentage of surviving mice on the total number of infected mice. The data were pooled from three independent experiments (n = 38 per strain).

SUPPLEMENTARY TABLES

TABLE S1. Cytokine/chemokine concentrations following chronic airway infection by wt and Δ*lasB* mutant RP45 strains. Concentrations in mice still infected at Day 7 post-infection (with a bacterial load in the lung ≥ 1000 colony forming units) are indicated. Statistical significance is indicated, and cytokines/chemokines/growth factors significantly different between the two strains are highlighted in grey.

Cytokine / Chemokine	Concentration (mean pg/700 µg lung protein ± SEM)	
	wt RP45 strain	Δ*lasB* RP45 strain
**IL-1α**	7.52 ± 1.18	16.51 ± 2.50**
**IL-1β**	3.16 ± 0.34	5.81 ± 0.60**
**IL-2**	6.26 ± 0.30	6.10 ± 0.30
**IL-3**	0.94 ± 0.05	1.14 ± 0.09
**IL-4**	0.53 ± 0.03	0.52 ± 0.04
**IL-6**	3.78 ± 0.06	3.96 ± 0.28
**IL-9**	18.92 ± 0.77	21.22 ± 0.96*
**IL-12p40**	34.37 ± 2.49	34.37 ± 2.62
**IL-12p70**	18.46 ± 1.23	28.89 ± 1.71**
**IL-17A**	11.53 ± 1.04	13.41 ± 1.91
**Eotaxin**	576.8 ± 24.36	633.3 ± 26.02
**G-CSF**	10.55 ± 0.99	22.82 ± 3.77**
**IFN-γ**	20.82 ± 0.81	23.24 ± 0.55
**KC**	65.13 ± 5.16	98.36 ± 18.00
**MCP-1**	170.1 ± 11.97	191.9 ± 9.42
**MIP-1α**	14.98 ± 2.57	46.10 ± 8.53***
**MIP-1β**	36.71 ± 3.97	87.29 ± 15.42**
**RANTES**	250.2 ± 23.04	583.3 ± 121.2*
**TNF-α**	31.97 ± 1.10	38.82 ± 1.57**

C57BL/6NCrlBR male mice (8 to 10 weeks old) were infected i.t. with an average of $5.6x10^{5}$ colony forming units of the *P. aeruginosa* wt and Δ*lasB* RP45 strains embedded in agar-beads. After seven days, murine lungs were collected, homogenized and centrifuged. The supernatants were used to measure cytokine/chemokine levels by Bio-Plex assay. Data are expressed as mean values ± standard errors of the mean (SEMs) of results from mice pooled from three independent experiments (n = 8-18). Statistical significance is indicated as follows: *, p<0.05; **, p<0.01; ***, p<0.001.

SUPPLEMENTARY REFERENCES

Bragonzi, A., Paroni, M., Nonis, A., Cramer, N., Montanari, S., Rejman, J., et al. (2009). Pseudomonas aeruginosa microevolution during cystic fibrosis lung infection establishes clones with adapted virulence. *American journal of respiratory and critical care medicine* 180(2), 138-145. doi: 10.1164/rccm.200812-1943OC.

Cigana, C., Lore, N.I., Riva, C., De Fino, I., Spagnuolo, L., Sipione, B., et al. (2016). Tracking the immunopathological response to Pseudomonas aeruginosa during respiratory infections. *Scientific Reports* 6, 21465. doi: 10.1038/srep21465.

Facchini, M., De Fino, I., Riva, C., and Bragonzi, A. (2014). Long term chronic Pseudomonas aeruginosa airway infection in mice. *Journal of visualized experiments : JoVE* (85). doi: 10.3791/51019.
